# First-in-class trispecific VHH-Fc based antibody with potent prophylactic and therapeutic efficacy against SARS-CoV-2 and variants

**DOI:** 10.1038/s41598-022-07952-4

**Published:** 2022-03-09

**Authors:** Allison Titong, Sachith Gallolu Kankanamalage, Jianbo Dong, Betty Huang, Nicholas Spadoni, Bo Wang, Meredith Wright, Keegan L. J. Pham, Anh Hai Le, Yue Liu

**Affiliations:** 1 Ab Studio Inc., 3541 Investment Blvd., Suite 3, Hayward, CA 94545 USA; 2 Ab Therapeutics Inc., 3541 Investment Blvd., Suite 2, Hayward, CA 94545 USA

**Keywords:** Antibody therapy, Antibody therapy, SARS-CoV-2, Drug development

## Abstract

SARS-CoV-2 and its variants have persisted in this ongoing COVID-19 pandemic. While the vaccines have greatly reduced the COVID-19 cases, hospitalizations, and death, about half of the world remain unvaccinated due to various reasons. Furthermore, the duration of the immunity gained from COVID-19 vaccination is still unclear. Therefore, there is a need for innovative prophylactic and treatment measures. In response to this need, we previously reported on the successful computer-aided development of potent VHH-based multispecific antibodies that were characterized in vitro. Here, we evaluated in vivo efficacy and safety of the lead trispecific VHH-Fc, ABS-VIR-001. Importantly, our data showed that ABS-VIR-001 treatment prevented SARS-CoV-2 infection and death when provided as an intranasal prophylaxis in a humanized ACE-2 mouse model. In addition, ABS-VIR-001 post-exposure treatment was shown to greatly reduce viral loads by as much as 50-fold. A detailed panel of metabolic and cellular parameters demonstrated that ABS-VIR-001 treatment was overall comparable to the PBS treatment, indicating a favorable safety profile. Notably, our inhibition studies show that ABS-VIR-001 continued to demonstrate unwavering efficacy against SARS-CoV-2 mutants, associated with key variants including Delta and Omicron, owing to its multiple epitope design. Lastly, we rigorously tested and confirmed the excellent thermostability of ABS-VIR-001 when heated to 45 °C for up to 4 weeks. Taken together, our study suggests that ABS-VIR-001 is an efficacious and durable prophylaxis and post-exposure treatment for COVID-19 with promising safety and manufacturability features for global distribution.

## Introduction

The coronavirus disease 2019 (COVID-19) pandemic has impacted human health worldwide, causing around 253 million infections and 5.1 million deaths (numbers based on statistics from November 16th, 2021)^1^. This disease is caused by the single stranded RNA β-coronavirus SARS-CoV-2^2,3^, which binds the receptor angiotensin converting enzyme-2 (ACE- 2) on human epithelial cells, becomes internalized, and replicates allowing the virus to spread^3^. In severe cases, the infection can cause lung damage and inflammatory hyperactivation, resulting in multiple organ failure and mortality^4^. In many countries, COVID-19 vaccination efforts have reduced infections and hospitalizations caused by the SARS-CoV-2. In the US, vaccines are the only FDA-approved form of prophylaxis against the viral infection to date. Despite this great medical feat, about half of the world is vaccinated and vulnerable to infection, due to lack of access, contraindications to the vaccine, or personal beliefs. Furthermore, long term vaccine immunity and vaccine efficacy against variants^5,6^ are still unknown potentially opening a window of opportunity for infection.

With limited availability of COVID-19 therapeutics that can prevent and/or treat SARS-CoV-2 infection, it is imperative to continue to develop preventative and treatment measures against SARS-CoV-2. In this manner, we previously reported the discovery of llama single domain antibodies (VHHs) that bind to SARS-CoV-2 spike (S) protein and block the binding of S protein to the human receptor ACE-2 screened from our robust in-house naïve and synthetic humanized llama VHH libraries^7,8^. Then, using computer-aided design, we rapidly and efficiently developed bispecific and trispecific antibodies based on anti-SARS-CoV-2 VHHs targeting multiple epitopes arranged in tandem and fused to human IgG1 Fc domain. This design allows for the capacity to withstand mutations against emerging viral variants and is equipped with multiple mechanisms of action^7,8^. Importantly, with computer-aided design, we were able to optimize the antibodies with enhanced safety, efficacy, and manufacturability balance. Our in vitro characterization demonstrated the potency of our multispecific antibodies, particularly of the trispecific antibodies which were more effective in both S binding and S/ACE-2 blocking compared to bispecific antibodies and monoclonal antibodies used in combination. In addition, the trispecific antibodies better blocked the SARS-CoV-2 pseudovirus from infecting human cells compared to a monoclonal antibody cocktail. We further showed that a lead trispecific antibody targets multiple epitopes in the S protein and has favorable biophysical characteristics for manufacturability ease^8^. Taken together, this lead antibody demonstrated high potency in vitro with promising manufacturability features.

In this study, we performed successful in vivo characterizations of safety and efficacy of the lead trispecific antibody ABS-VIR-001 (also known as 1B-3F-2A-Fc). We also demonstrated excellent thermostability of ABS-VIR-001 (derived from VHH, well-known for its natural thermostability^9–11^), which is a key component for global distribution and storage, further contributing to a promising manufacturability campaign. Lastly, we evaluated the durability of ABS-VIR-001 against mutants associated with the key variants of the pandemic, including Alpha, Beta, Delta, Lambda, and Omicron. Ultimately, our results suggest that ABS-VIR-001 can be further developed as a clinically relevant prophylactic and therapeutic drug against SARS-CoV-2 and the emerging viral variants.

## Materials and methods

### Materials

The SARS-CoV-2 pseudovirus was obtained from (GENEWIZ, Batch Number: PSEUV02). The SARS-CoV-2 authentic virus was obtained from (Harbin Veterinary Research Institute, Batch Number: SARS-CoV-2/HRB24/human/2020/CHN). The recombinant SARS-CoV-2 S wild-type (Cat.# SPN-C52H9-50ug), SARS-CoV-2 Delta (Cat.# SPN-C52He-50ug), SARS-CoV-2 Lambda (Cat.# SPN-C52Hs-50ug), SARS-CoV-2 Omicron (Cat.# SPN-C52Hz), ACE-2 (Cat.# AC2-H52H8-50ug), biotinylated ACE-2 (ACE-2-b) (Cat.# AC2-H82E6-200ug) were obtained from ACROBiosystems. The mFc-tagged ACE-2 (Cat.# 10,108-H05H) and SARS-CoV-2 S Omicron (Cat.# 40,589-V08H26) was obtained from SinoBiologicals.

### Cell transfection, antibody expression, and antibody purification

The Expi293F cells (Thermo Fisher Scientific) were cultured in a humidified chamber at 37 °C, 5% CO_2_ in Expi293 expression medium (Thermo Fisher Scientific). They were transiently transfected with plasmids encoding antibodies using ExpiFectamine 293 transfection reagent (Thermo Fisher Scientific) as instructed by the manufacturer and stated in detail in our previous publication^8^. Briefly, the Expi293F cells were plated in fresh media at a density of 1.7 × 10^6^ cells/ml and cultured overnight. The next day, DNA and ExpiFectamine reagent were separately mixed in Opti-MEM, and incubated at room temperature for 3 min, and combined. After further incubation for 20 min at room temperature, it was added to cells followed by the addition of enhancers at 17 h after transfection. The cells were harvested 72 h after transfection, the cell supernatant was passed through a 0.45 µm membrane, and the antibody concentration was measured by biolayer interferometry using GatorPrime (Gator Bio) using a Protein A probe. The antibodies were purified by Protein A columns in an AKTA Explorer 100 purification system, dialyzed twice in PBS, and finally passed through a 0.22 µm membrane.

### Animal studies

All animal studies were performed in contract with Shanghai Model Organisms Center, Inc.

#### Pseudovirus infection study

6–8-week-old female Ubc-CreER x Rosa-CAG-LSL-ACE2-IRES-tdTomato mice, weighing ~ 20–30 g, were used for this study. A total of 11 mice were obtained from Shanghai Model Organisms Center, Inc. Animals were housed in a specific pathogen free (SPF) laboratory animal room and provided food and water ad libitum. To induce the expression of huACE-2, the mice were administered with 100 mg/kg tamoxifen by intraperitoneal (I.P.) injection five times from pre-inoculation day 11 to 3, on every other day. Three days after the last tamoxifen dose (day 0), mice were intranasally (I.N.) challenged with 6.05 × 10^4^ transducing units (TU) of SARS-CoV-2-luc pseudovirus expressing luciferase at Day 0 (D0). Mice were then randomly divided into 3 groups. No treatment was provided to the control group of 4 mice (Group 1). The treatment groups were treated with 10 mg/kg of ABS-VIR-001 by I.N. administration at 10 h pre-infection (-10 hpi, Group 2, prophylaxis model) of 3 mice or 2 h post-infection (+ 2 hpi, Group 3, post-exposure treatment model) of 3 mice, respectively. The body weights of mice were monitored daily and bioluminescence (BLI) measurements of SARS-CoV-2-luc were obtained on D3, D4 and D7.

#### Authentic SARS-CoV-2 infection study

6–8-week-old male NM-KI-200272 CAG-human ACE2-IRES-Luciferase-WPRE-polyA mice, weighing ~ 20–30 g, were used for this study. A total of 22 mice were obtained from Shanghai Model Organisms Center, Inc. Animals were housed in a SPF laboratory animal room in a Biosafety Level 3 Laboratory of Harbin Veterinary Research Institute and provided food and water ad libitum. The mice were I.N. challenged with 1000 PFU of SARS-CoV-2 (SARS-CoV-2/HRB24/human/2020/CHN) at D0. Then, the mice were randomly divided into 4 groups. No treatment was provided to the control group of 5 mice (Group 1). The treatment groups were treated with 25 mg/kg of ABS-VIR-001 by I.N. administration at 10 h pre-infection (-10 hpi, Group 2, prophylaxis model) of 5 mice or 2 h post-infection (+2 hpi, Group 3, post-exposure treatment model) of 6 mice, or with 10 mg/kg of ABS-VIR-001 by intraperitoneal (I.P.) administration 2 h post-infection (+ 2 hpi, Group 4, post-exposure treatment model) of 6 mice. The body weights and survival of mice were monitored daily and after D4 (D3 if the mouse succumbed to infection sooner), the lung was harvested and measured for SARS-CoV-2 titers using rt-PCR.

#### ABS-VIR-001 safety study

6–8-week-old female M-NSG mice weighing ~ 17–23 g was used for this study. A total of 16 mice were obtained from Shanghai Model Organisms Center, Inc. Animals were housed in a SPF laboratory animal room and provided food and water ad libitum. The mice were randomly divided into 4 groups. PBS was provided I.N. and intravenously (I.V.) to the control group of 4 mice (Group 1). The treatment groups were treated with 25 mg/kg of ABS-VIR-001 by I.N. administration (Group 2) of 4 mice, 10 mg/kg of ABS-VIR-001 by I.N. administration (Group 3) of 4 mice, or 10 mg/kg of ABS-VIR-001 by I.V. administration (Group 4) of 4 mice. The body weights of mice were monitored and after D6, the mice were euthanized, and the blood was analyzed for hematological and metabolic parameters.

### Antibody thermostability analysis

The thermostability of ABS-VIR-001 was assessed by heating the antibody to 45º C for up to four weeks. Each week, a sample of the heated ABS-VIR-001 sample was analyzed by differential scanning fluorimetry (DSF) method using the UNcle system (Unchained Labs) v4.01 (https://www.unchainedlabs.com/uncle/). For the DSF assay, the temperature was increased at 1 °C/min from 25 °C to 95 °C to obtain the melting temperature (Tm) of the sample. The data were analyzed and calculated by the UNcle Analysis Software. The binding kinetics of heated ABS-VIR-001 were also analyzed using biolayer interferometry, weekly.

### Biolayer interferometry

Biolayer interferometry assays were performed to assess quantitative and qualitative ABS-VIR-001 binding using the GatorPrime system (Gator Bio) v2.7.3.0728 (https://www.gatorbio.com/).

#### Quantitative ABS-VIR-001 concentration assessment

A glass Protein A probe (Gator Bio) was dipped in purified ABS-VIR-001. Then, the binding signal was analyzed against an in-house Protein A-based standard curve and calculated by the GatorPrime (Gator Bio) software.

#### Quantitative ABS-VIR-001 binding kinetics

A glass hFC probe (Gator Bio) was initially dipped into Q Buffer (Gator Bio) for about 120 s yielding the baseline signal. Then the probe was loaded with 3–5 µg/ml of ABS-VIR-001 diluted Q Buffer for about 200 s and followed by a Q Buffer wash step of about 120 s. Next, the bound ABS-VIR-001 was associated with 100–3.125 nM of S protein (wild-type or mutants), including a no S protein control (0 nM) for about 180 s followed by a dissociation step of up to 900 s. The association and dissociation curves were graphed and calculated by the GatorPrime (Gator Bio) software to yield binding kinetics values (koff, Kon, and KD).

#### Qualitative ABS-VIR-001 binding and inhibition

A glass streptavidin probe (Gator Bio) was initially dipped into Q Buffer (Gator Bio) for about 120 s yielding the baseline signal. Then the probe was loaded with 5 µg/ml of ACE-2-b diluted in Q Buffer for about 60–120 s and followed by a Q Buffer wash step of about 120 s. Next, the bound ACE-2-b was associated with a 1:5 ratio or 1:20 ratio S protein (wild-type or mutants) to ABS-VIR-001 pre-mixture, including a no ABS-VIR-001 control (0 nM) for about 180 s followed by a dissociation step of up to 300 s. The association curves were graphed by the GatorPrime (Gator Bio) software to yield qualitative binding and inhibition signals in the absence or presence of ABS-VIR-001.

### ELISA

High-binding 96-well plates were coated overnight with 1 µg/ml of SARS-CoV-2 S RBD at 4ºC. ABS-VIR-001 was prepared at 125 µg/ml and serially diluted fivefold and the three VHH-Fcs were prepared for the VHH-cocktail at 75 µg/ml of each and serially diluted fivefold in ELISA assay buffer consisting of 1 × PBS with 1% (w/v) BSA. After washing and blocking the plate, antibody dilutions were added and incubated for 30–45 min at RT with shaking. The plate was washed again and treated with goat anti-human Fc-HRP for an additional 30–45 min at RT with shaking. Finally, the plate was washed and treated with development buffer containing Amplex Red and H_2_O_2_ before detecting emitted fluorescent signal from each well on a SpectraMax Gemini XPS plate reader using SoftMaxPro v5.4 to detect binding. In contrast, if blocking was assessed, 0.45 ug/ml of ACE-2-b was added in lieu of anti-human Fc-HRP, followed by a Strep-HRP detection antibody, and emitted fluorescent signals were read using the SpectraMax Gemini XPS plate reader using SoftMaxPro v5.4.

### Data analysis

The data was analyzed by the software Prism (GraphPad) v9.2.0 (https://www.graphpad.com/). Any statistical analysis performed are indicated in the figure legends.

### Ethics statement

The mouse experiments were approved by the Institutional Animal Care and Use Committee of Harbin Veterinary Research Institute and performed in accordance with institutional guidelines. The studies were performed in compliance with the ARRIVE guidelines.

## Results

### ABS-VIR-001 prevents SARS-CoV-2-luc pseudovirus infection in huACE-2 mice

**Figure 1:**
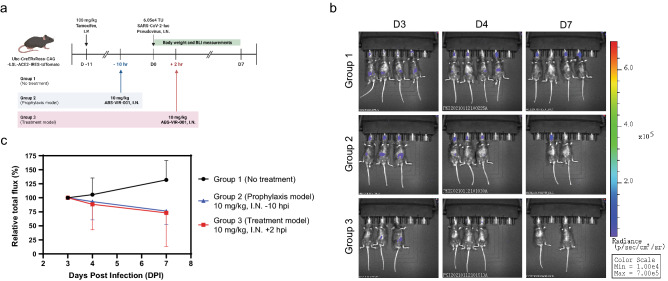
ABS-VIR-001 prevents SARS-CoV-2 pseudovirus infection in huACE-2 mice. Tamoxifen inducible ACE-2 mice were challenged with SARS-CoV-2-luc pseudovirus (intranasally, I.N.) and were randomly divided into 3 groups and treated as indicated and observed for body weight and bioluminescence (BLI). (**A**) Workflow of the pseudovirus animal study. (**B**) The bioluminescent imaging from D3, D4, and show drastic BLI reduction from D3 to D7 for Group 2 and Group 3, in contrast to the no treatment Group 1. (Error bars = SD) The graph was generated using the Prism (GraphPad) software v9.2.0 (https://www.graphpad.com/). (**C**) The relative total flux percentage of the quantified BLI measurements show a decreasing trend for Group 2 and Group 3, in contrast to the negative control Group 1.

Initially, ABS-VIR-001 was evaluated in a SARS-CoV-2-luc pseudovirus challenge model using an inducible human ACE-2 (huACE-2) mouse (Fig. 1a). After the expression of huACE-2 was induced, mice were divided into three groups; a no treatment group (Group 1), a prophylaxis group (Group 2, intranasal treatment of 10 mg/kg ABS-VIR-001 10 h prior to virus challenge), and a post-exposure treatment group (Group 3, intranasal treatment of 10 mg/kg ABS-VIR-001 2 h post virus challenge). At day 0 (D0), the mice were challenged intranasally with the virus and monitored for body weight daily from D0 to D7 (Figure S1a) and BLI imaging and measurements from D3, D4, and D7 (Fig. 1b,c, respectively). The body weights of the antibody-treated animals were comparable to the no treatment control (Figure S1a), indicating that ABS-VIR-001 is well-tolerated. Importantly, the relative total flux percentage from the BLI imaging and measurements clearly decreased in trend for both the prophylaxis model and treatment model in contrast to the no treatment group (Fig. 1b,c), which was initially more dramatic for the prophylaxis group (Figure S1b). Overall, this data suggests that ABS-VIR-001 is highly efficacious as a prophylaxis and as a post-exposure treatment against the SARS-CoV-2-luc pseudovirus via the intranasal route.

### ABS-VIR-001 prevents SARS-CoV-2 infection and decreases viral burden after infection in huACE-2 mice

**Figure 2:**
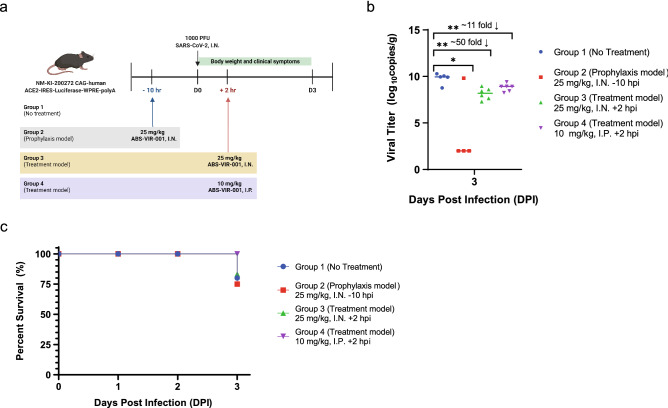
ABS-VIR-001 prevents SARS-CoV-2 infection and death and decreases viral burden in huACE-2 mice challenged with authentic SARS-CoV-2. Human ACE-2 expressing mice were challenged with SARS-CoV-2 (intranasally, I.N.) and were randomly divided into 4 groups and treated as indicated and observed for body weight and clinical symptoms. (**A**) Workflow of the SARS-CoV-2 animal study. B) On D3, the lungs of the mice were harvested and viral titers were assessed by RT-qPCR. Statistical significance was calculated by a student t-test and a *P* value of < 0.05 was considered statistically significant. This data shows that viral titer was not detected (3 out of the 4 mice in the prophylaxis Group 2) or significantly decreased in all mice treated with ABS-VIR-001, indicating that ABS-VIR-001 is a potent prophylactic and treatment for SARS-CoV-2 infection. (**C**) After D3, all mice within the I.P. Treatment group survived (Group 4) or showed similar survival (Groups 2 and 3) as the no treatment group (Group 1), indicating that ABS-VIR-001 is well-tolerated and more importantly, ABS-VIR-001 prevented death. The graphs were generated using the Prism (GraphPad) software v9.2.0 (https://www.graphpad.com/).

Based on the success of the pseudovirus study, we next assessed the efficacy of ABS-VIR-001 on an authentic SARS-CoV-2 challenge model using mice constitutively expressing huACE-2 (Fig. 2a). First, the appropriate virus inoculation dose was assessed in this mouse model (Table S1). In this initial analysis, it was determined that 1000 PFU established the most consistent and high titers within the group in comparison to the other inoculation groups by D3 (Table S2). Furthermore, mice inoculated at the lower doses already displayed an increase in pathological changes from 10 to 100 PFU in the mouse lung tissue (Figure S2a and Table S3). Taken together, a new set of mice were divided into four groups (Fig. 2a); a no treatment group (Group 1), an I.N. prophylaxis group (Group 2, at 25 mg/kg ABS-VIR-001 10 h prior to virus challenge), an I.N. treatment group (Group 3, at 25 mg/kg ABS-VIR-001 2 h post virus challenge) and an I.P. treatment group (Group 4, at 10 mg/kg ABS-VIR-001 2 h post virus challenge). At day 0 (D0), the mice were challenged with 1000 PFU of SARS-CoV-2 virus and monitored for body weight daily from D0 to D3 (Figure S3a) and lungs were harvested for viral titer measurements on D3 (Fig. 2b). No weight loss was observed in the I.N. prophylaxis group in all the animals within the group, and the body weights of the other groups were comparable to the no treatment control group (Figure S3a and Table S1), indicating the safety of ABS-VIR-001 for both the I.N. and I.P. route. Importantly, the lung viral titer measurements decreased for both the prophylaxis model and treatment models in contrast to the no treatment group (Fig. 2b), which was most significant for the I.N. for the prophylaxis group with 3 out of the 4 animals showing undetectable levels of virus and the treatment models with about 11- or 50- fold reduction in virus in comparison to the no treatment group. After D3, all mice within the I.P. Treatment group survived (Group 4) or showed similar survival (Groups 2 and 3) as the no treatment group (Group 1), indicating that ABS-VIR-001 is well-tolerated and more importantly, ABS-VIR-001 prevented death (Fig. 2c). This data suggests that ABS-VIR-001 is highly efficacious as a prophylaxis treatment and as a treatment for SARS-CoV-2 via the I.N. and I.P. (surrogate for the I.V.) route.

### ABS-VIR-001 does not cause significant changes in safety biomarkers in immunodeficient mice

**Figure 3:**
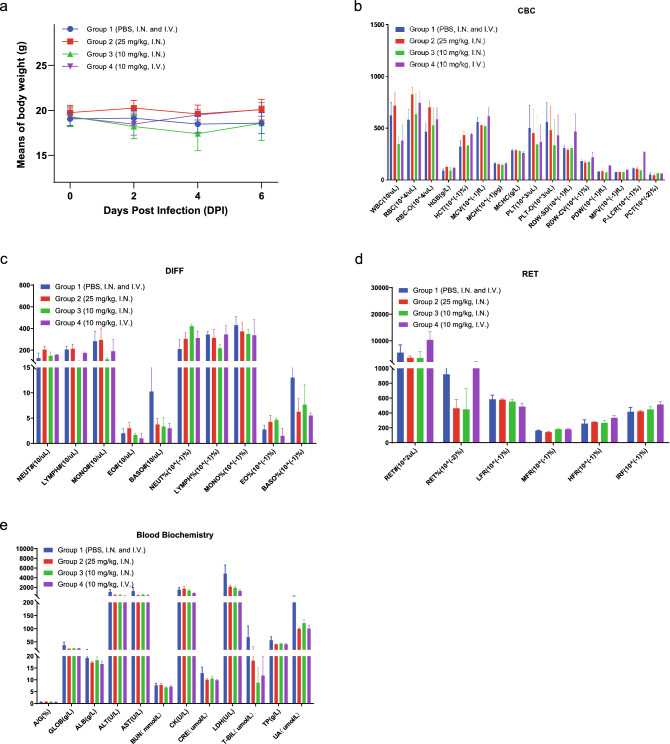
ABS-VIR-001 is safe in animals based on a blood biomarker assessment. M-NSG mice were divided into 4 treatment groups as indicated and observed for body weights until D6. Then, the mice were euthanized, and blood was collected for hematological and metabolic evaluation. Overall, based on all the blood parameters evaluated, no major differences in were observed between ABS-VIR-001 treated mice and PBS control treated mice, indicating that ABS-VIR-001 is safe. (**A**) The quantitation of the mouse bodyweights over time following antibody treatment, represented by mean body weight per treatment group. B) Complete blood count (CBC) panel. (**C**) Blood differential (DIFF) panel. (**D**) Reticulocyte (RET) count. (**E**) Blood biochemistry panel. The graph was generated using the Prism (GraphPad) software v9.2.0 (https://www.graphpad.com/).

We previously observed that a mouse from Group 2 and a mouse from Group 3 were found dead on D7 in the initial SARS-CoV-2-luc pseudovirus infection study. In this regard, we carried out an in vivo safety study of ABS-VIR-001 on immunodeficient mice to rule out any safety concerns associated with ABS-VIR-001. Mice were divided into four groups and at D0, each mouse was treated in the following manner: a PBS group (Group 1, PBS I.N. and I.V.), an I.N. group (Group 2, at about 25 mg/kg ABS-VIR-001), an I.N. group (Group 3, at 10 mg/kg ABS-VIR-001) and an I.V. treatment group (Group 4, at 10 mg/kg ABS-VIR-001). Body weight was monitored every other day 15 days prior to the treatment start day (D0) until D6. After D6, hematologic and metabolic parameters were assessed to determine potential negative effects. Consistent with previous data, the bodyweight of the mice only showed minor variations among groups after the treatment, overall suggesting that ABS-VIR-001 is well-tolerated by the animals (Fig. 3a). The assessment of the hematologic parameters between all treatment groups showed no major differences compared to the control group (Fig. 3b,c). The WBC for the 10 µg/kg I.N. and I.V. groups were slightly decreased. RDW, PDW, and P-LRC% for the 10 µg/kg I.V. group were slightly elevated. The RET% for the 10 µg/kg I.N. and 25 µg/kg I.N. groups were slightly decreased (Fig. 3d). As shown in Fig. 3e, no major differences were also observed in the metabolic parameters in treatment groups compared to the control group. The LDH, T-BIL, and UA for the 10 µg/kg I.N., 25 µg/kg I.N and 10 µg/kg I.V. groups were slightly decreased. Taken together, this data suggests that ABS-VIR-001 is safe in animals.

### ABS-VIR-001 has high thermostability

**Figure 4:**
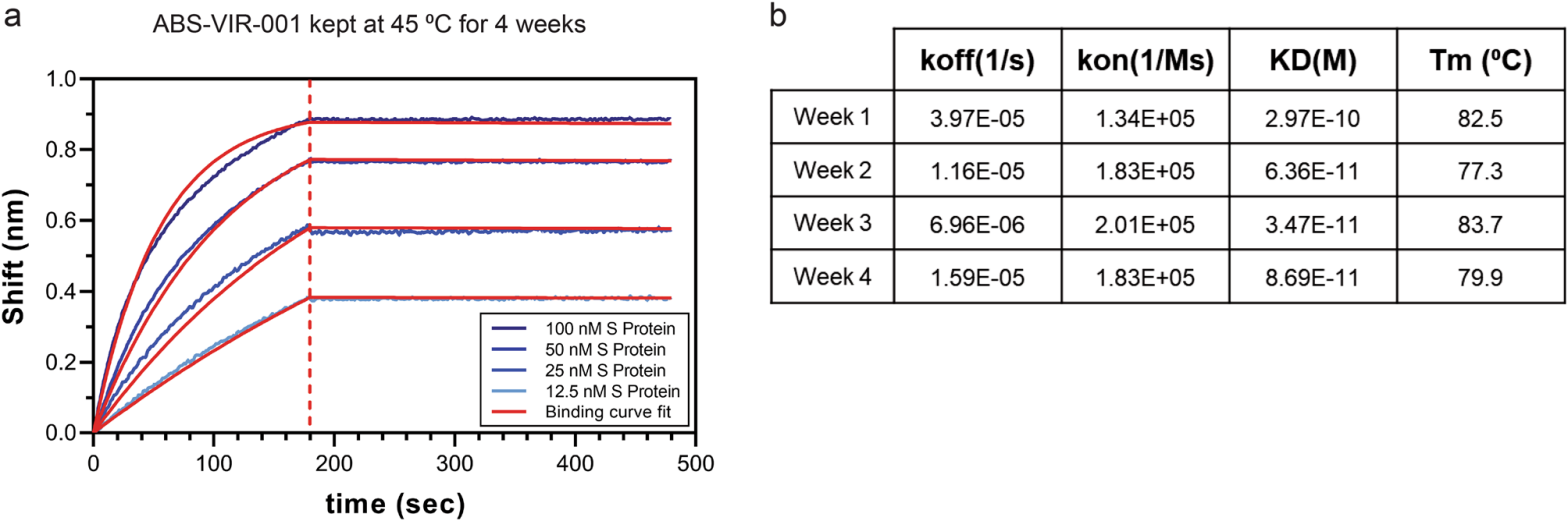
ABS-VIR-001 has high thermostability and aggregation resistance. (**A**) Binding kinetics graph for global KD after ABS-VIR-001 was kept at 45 °C for 4 weeks. The data was generated by the GatorPrime software (Gator Bio) v 2.7.3.0728 (https://www.gatorbio.com/), and graphed in Prism (GraphPad) software v9.2.0 (https://www.graphpad.com/). (**B**) The weekly changes in biophysical characteristics of ABS-VIR-001 over time. The koff, kon, and KD values were calculated by the GatorPrime software following biolayer interferometry analysis by GatorPrime. The Tm values were calculated by the UNcle software (Unchained Labs) v4.01 (https://www.unchainedlabs.com/uncle/) following DSF analysis by UNcle. Taken together, this data shows the favorable features conducive for manufacturability and global distribution.

Previously we showed that our bispecific and trispecific VHH-Fcs including ABS-VIR-001 have favorable biophysical characteristics^8^. Here, we further demonstrated the thermostability of ABS-VIR-001 by subjecting the antibody to 45 °C for 4 weeks and assessing binding kinetics (GatorPrime biolayer interferometry) and biophysical features (DSF method) weekly. Consistent with our previous data, ABS-VIR-001 displayed stable binding to SARS-CoV-2 S protein even after 4 weeks at 45 °C (Fig. 4a,b). Furthermore, the melting temperature (Tm) measurement for each week showed little change throughout the 4-week study, indicating high thermostability (Fig. 4b). Based on these results, ABS-VIR-001 shows suitability for large-scale manufacturing and stability of the antibody in a high temperature situation, especially in global distribution and storage situations.

### ABS-VIR-001 binds and blocks the ACE-2 interaction of SARS-CoV-2 mutants

One of the hallmarks of our trispecific VHH-Fc is the ability bind its antigen using multiple epitopes^8^. We previously theorized that this multi-epitope design of ABS-VIR-001 can antagonize viral mutation escape so that even if mutations in one epitope causes loss of binding to one VHH, other VHHs can maintain binding to the unmutated epitopes^8^. Thus, ABS-VIR-001 is a feasible treatment approach to prevent and treat emerging SARS-CoV-2 variants^3,4^. In this manner, we tested the ability of ABS-VIR-001 to bind the S protein and block the S/ACE-2 interaction among mutant S proteins corresponding to major SARS-CoV-2 variants of concern from this pandemic using ELISA or GatorPrime biolayer interferometry methods. First, we tested the binding of ABS-VIR-001 to the receptor binding domain (RBD) of an S protein with the mutations K417N, E484K, and N501Y (Tri-mutant). These three mutations are associated with the Beta SARS-CoV-2 variant whereas the mutation N501Y is also associated with the Alpha SARS-CoV-2 variant^4,12,13^. As shown in Figure S4a, ABS-VIR-001 maintained strong binding via biolayer interferometry to the Tri-mutant with a KD of 2.49E-10 M (Figure S4b). More importantly, ABS-VIR-001 showed similar blocking of S/ACE-2 interaction in the presence of both wild-type S RBD and Tri-mutant S RBD via ELISA (Figure S5a), suggesting that it would maintain similar efficacy against wild-type and variant SARS-CoV-2. In addition, ABS-VIR-001 was more efficacious at blocking both wild-type S RBD and Tri-mutant S RBD interactions with ACE-2 compared to a VHH-Fc cocktail comprising of its individual subunits, further demonstrating the superiority of the multispecific antibody format over the antibody combination approach (Figure S5a).

**Figure 5:**
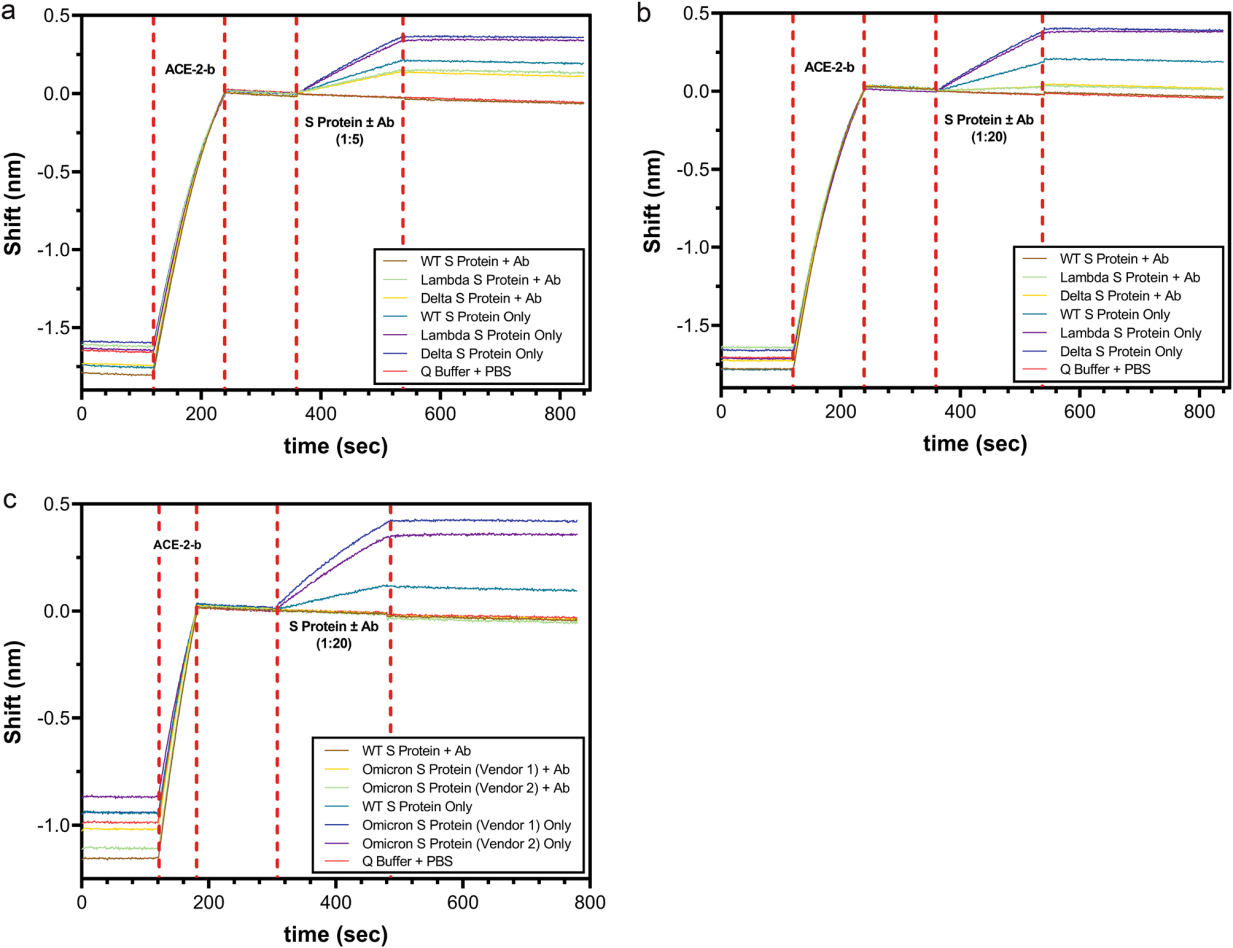
ABS-VIR-001 effectively blocks SARS-CoV-2 Delta and Lambda variants. Blocking of ABS-VIR-001 were assessed using the GatorPrime biolayer interferometry machine and software. Briefly, a streptavidin sensor was loaded with biotinylated ACE-2 (ACE-2-b) and then dipped in S protein trimers (wild-type, Delta, Lambda, or Omicron) in the absence or presence of ABS-VIR-001. In the absence of ABS-VIR-001, Delta, Lambda, and Omicron S binds ACE-2 with a higher binding signal than wild-type S (green blue), indicating that much more antibody maybe necessary to completely block these variants. (**A**) At a 1:5 ratio of S to ABS-VIR-001 (Ab), there was complete blocking of wild-type S (red) and significant, but partial blocking in Delta (orange), and Lambda S (green). Interestingly, the overall blocking potential at this treatment is similar to the wild-type S, suggesting that the antibody affinity to the variants is similar to wild-type. (**B**) A higher S to ABS-VIR-001 (Ab) ratio of 1:20, resulted in nearly complete blocking of Delta and Lambda variants. (**C**) S to ABS-VIR-001 (Ab) ratio of 1:20 resulted in complete blocking of Omicron variant S trimers obtained from two different vendors. The data was generated by the GatorPrime software (Gator Bio) v2.7.3.0728 (https://www.gatorbio.com/), and graphed in Prism (GraphPad) v9.2.0 (https://www.graphpad.com/). Taken together, ABS-VIR-001 demonstrates unwavering efficacy against key variants throughout the pandemic.

We also tested the efficacy of ABS-VIR-001 in blocking the S/ACE-2 interactions associated with the S mutants associated with Delta and Lambda SARS-CoV-2 variants^4,12–14^. As shown in Fig. 5, the binding and blocking of ABS-VIR-001 to Delta, Lambda, or Omicron S were assessed by GatorPrime biolayer interferometry. Interestingly, in the absence of ABS-VIR-001, the S associated with Delta, Lambda (Fig. 5a,b), and Omicron (Fig. 5c) variants bound the ACE-2 much more than the wild-type. In the presence of ABS-VIR-001, ACE-2 binding was greatly reduced for Delta S and Lambda S and completely blocked for wild-type S (Fig. 5a). Since there is a much greater binding between the Delta and Lambda S to ACE-2 in comparison to the wild-type S, we reasoned that more ABS-VIR-001 was necessary to further block the S/ACE-2 interaction for these variants. To this regard, Fig. 5b demonstrated that the increase in ABS-VIR-001 did lead to a greater degree of blocking of Delta and Lambda S binding to ACE-2. Following the emergence of a new variant, our recent data shows that ABS-VIR-001 also completely blocks the Omicron S/ACE-2 interaction (Fig. 5c). Ultimately, these results suggest that ABS-VIR-001 has great potential to prevent and treat infections caused by emerging SARS-CoV-2 variants.

## Discussion

The COVID-19 pandemic caused by the SARS-CoV-2 has become a severe public health crisis with limited treatment options even after more than two years since its emergence^1,2^. Vaccination is currently the best form of protection against the virus, however certain fractions of the population remain unvaccinated and there is evidence that the vaccines may have diminished efficacy against emerging SARS-CoV-2 variants^5,6^. These facts highlight the need for developing novel agents with prevention and/or treatment capabilities against SARS-CoV-2. In this sense, we have developed the tri-specific VHH-Fc antibody ABS-VIR-001 as a dual action candidate against SARS-CoV-2, both as a potential prophylactic and drug.

Several antibody treatments have received the emergency use authorization (EUA) by the Food and Drug Administration (FDA) to treat COVID-19^15^. Among them, REGEN-COV (Casirivimab and Imdevimab) (Regeneron) and Bamlanivimab and Etesevimab (Eli Lilly) act as monoclonal antibody cocktails, whereas Sotrovimab (GSK) acts as a single agent monoclonal antibody against SARS-CoV-2^15–19^. In addition, Evusheld (Tixagevimab and Cilgavimab) (AstraZeneca) have been approved under the EUA as an anti-SARS-CoV-2 monoclonal antibody cocktail prophylactic^15,20^. These therapeutic/prophylactic antibodies contain the IgG format, as opposed to the VHH-Fc format contained by ABS-VIR-001. However, it is important to note that VHH antibodies (nanobodies) are becoming a widely researched and developed modality in the field of antibody therapeutics^21,22^. In fact, there are several potential VHH-based antibody therapies/prophylactics from camelid species currently being tested and developed against SARS-CoV-2, highlighting the feasibility of VHH antibodies as COVID-19 therapeutics and prophylactics^23–29^.

ABS-VIR-001 offers high efficacy as it binds to multiple epitopes within the SARS-CoV-2 S1 protein and contains Fc effector functions such as antibody-dependent cellular cytotoxicity (ADCC). It is more efficacious than using an antibody cocktail composed of individual monoclonal antibodies^8^. This feature could potentially make ABS-VIR-001 more potent than the currently available antibody cocktail therapies against SARS-CoV-2. This is exemplified by the reduction of SARS-CoV-2 infection in both pseudovirus and authentic virus animal models by ABS-VIR-001, especially regarding its preventative actions. It is interesting to note that while ABS-VIR-001 prevented the authentic SARS-CoV-2 infection to undetectable levels in the Prophylaxis group (Fig. 2b), one animal did not display reduction of the viral burden which could be due to technical issues of intranasal antibody administration. We also show that ABS-VIR-001 is well-tolerated and has no major negative hematological or metabolic effects in multiple in vivo models, suggesting its overall safety. These data indicates that ABS-VIR-001 would be safe in humans.

ABS-VIR-001 has high stability with favorable biophysical characteristics, showing that it is suitable for higher temperature storage and distribution. This would overcome one of the major barriers associated with current vaccines and would be ideal to be used in developing countries. In addition, ABS-VIR-001 has higher manufacturability and can be expressed in high concentrations in research level conditions (data not shown), suggesting that it has features for large-scale industrial production. In addition, we show that ABS-VIR-001 is amenable to I.N. administration. Intranasal vaccines have been recently developed against COVID-19 and constitutes a promising approach due to the feasibility of developing at-home prophylaxis and treatment options^30,31^. Similarly, ABS-VIR-001 has the potential to become an at-home I.N. prophylaxis/treatment option (such as a nasal spray) that can be self-administered by people with prior COVID-19 exposure (ex: immediate family members and close contacts of COVID-19 patients), patients with mild COVID-19 (ex: asymptomatic patients), and people with increased risk for future COVID-19 exposure (ex: people who travel and attend large gatherings). With these favorable features, ABS-VIR-001 is a suitable candidate for further clinical development.

Importantly, we also explored the landscape of SARS-CoV-2 variants in the context of ABS-VIR-001. Due to its multiple epitope binding, ABS-VIR-001 is more resistant to the viral antibody escape through mutations as a single mutation in the S1 structure would not completely lose its binding of ABS-VIR-001^8^. This would make it ideally suited to counter the emerging SARS-CoV-2 variants with different mutations. As we show in this study and summarized in Table 1, ABS-VIR-001 maintains its efficacy against mutants representing Delta, Lambda and Omicron variants, as well as Alpha and Beta variants.

**Table 1:** Summary of ABS-VIR-001 potency against key variants throughout the pandemic.

	**Binding**	**Blocking**
Wild-type	+ + +	+ + +
Other variants (Alpha/Beta)	+ + + / + +	+ + + / + +
Delta/Lambda	+ +	+ +
Omicron	+ + + / + +	+ + +

ABS-VIR-001 was developed by the computer-aided antibody design (CAAD)^7,8^. This technology platform allows for faster screening of antibody candidates with higher probability of future clinical success. In addition, it allows the proper optimization of candidates to have a high efficacy, safety, and manufacturability balance. This is exemplified by the fast development of ABS-VIR-001 as we have demonstrated before^7,8^, and favorable safety and manufacturability features we show in this study. This serves as a proof of the versatility of this technology platform for the rapid production of many types of therapeutic antibodies. This would be especially useful for anti-COVID-19 antibodies because when novel SARS-CoV-2 variants emerge, this platform has the potential to quickly develop antibodies by screening for novel VHH blockers and replacing VHH modules in the existing antibody molecules, so that therapeutic antibody production process can maintain pace with novel variant emergence.

## Supplementary Information

Supplementary Information.

## Data Availability

The data from this study is available from the corresponding author upon reasonable request.

## References

[1] *WHO Coronavirus (COVID-19) Dashboard*, <https://covid19.who.int/> (

[2] Hu B, Guo H, Zhou P, Shi ZL (2021). Characteristics of SARS-CoV-2 and COVID-19. Nat. Rev. Microbiol.

[3] Haque SM, Ashwaq O, Sarief A, Azad John Mohamed AK (2020). A comprehensive review about SARS-CoV-2. Future Virol..

[4] Cascella, M., Rajnik, M., Aleem, A., Dulebohn, S. C. & Di Napoli, R. in *StatPearls* (2021). 32150360

[5] Kathryn, M., Edwards, W. A. O. *COVID-19: Vaccines to prevent SARS-CoV-2 infection*, <https://www.uptodate.com/contents/covid-19-vaccines-to-prevent-sars-cov-2-infection#H2645692315> (2021).

[6] Tregoning JS, Flight KE, Higham SL, Wang Z, Pierce BF (2021). Progress of the COVID-19 vaccine effort: viruses, vaccines and variants versus efficacy, effectiveness and escape. Nat. Rev. Immunol.

[7] Dong J (2020). Development of multi-specific humanized llama antibodies blocking SARS-CoV-2/ACE2 interaction with high affinity and avidity. Emerg. Microbes Infect..

[8] Dong J (2020). Development of humanized tri-specific nanobodies with potent neutralization for SARS-CoV-2. Sci. Rep..

[9] Dumoulin M (2002). Single-domain antibody fragments with high conformational stability. Protein Sci..

[10] Arbabi Ghahroudi M, Desmyter A, Wyns L, Hamers R, Muyldermans S (1997). Selection and identification of single domain antibody fragments from camel heavy-chain antibodies. FEBS Lett..

[11] van der Linden RH (1999). Comparison of physical chemical properties of llama VHH antibody fragments and mouse monoclonal antibodies. Biochim Biophys Acta..

[12] Khateeb J, Li Y, Zhang H (2021). Emerging SARS-CoV-2 variants of concern and potential intervention approaches. Crit. Care.

[13] Krause PR (2021). SARS-CoV-2 Variants and Vaccines. N. Engl. J. Med..

[14] Kimura, I. *et al.* SARS-CoV-2 Lambda variant exhibits higher infectivity and immune resistance. *bioRxiv*, 2021.2007.2028.454085, 10.1101/2021.07.28.454085 (2021).

[15] *Coronavirus Disease 2019 (COVID-19) EUA Information*, <https://www.fda.gov/emergency-preparedness-and-response/mcm-legal-regulatory-and-policy-framework/emergency-use-authorization#coviddrugs> (

[16] Hansen J (2020). Studies in humanized mice and convalescent humans yield a SARS-CoV-2 antibody cocktail. Science.

[17] Copin R (2021). The monoclonal antibody combination REGEN-COV protects against SARS-CoV-2 mutational escape in preclinical and human studies. Cell.

[18] Gottlieb RL (2021). Effect of Bamlanivimab as monotherapy or in combination with Etesevimab on viral load in patients with mild to moderate COVID-19: A randomized clinical trial. JAMA.

[19] Gupta A (2021). Early treatment for Covid-19 with SARS-CoV-2 neutralizing antibody sotrovimab. N. Engl. J. Med..

[20] *Evusheld (formerly AZD7442) long-acting antibody combination authorised for emergency use in the US for pre-exposure prophylaxis (prevention) of COVID-19*, <https://www.astrazeneca.com/media-centre/press-releases/2021/evusheld-long-acting-antibody-combination-authorised-for-emergency-use-in-the-us-for-pre-exposure-prophylaxis-prevention-of-covid-19.html> (2021).

[21] Steeland S, Vandenbroucke RE, Libert C (2016). Nanobodies as therapeutics: big opportunities for small antibodies. Drug Discovery Today.

[22] Bathula NV, Bommadevara H, Hayes JM (2021). Nanobodies: The future of antibody-based immune therapeutics. Cancer Biother. Radiopharm..

[23] Lu Q (2021). Development of multivalent nanobodies blocking SARS-CoV-2 infection by targeting RBD of spike protein. J. Nanobiotechnol..

[24] Schepens, B. *et al.* An affinity-enhanced, broadly neutralizing heavy chain–only antibody protects against SARS-CoV-2 infection in animal models. *Sci. Transl. Med.* **13**, eabi7826, 10.1126/scitranslmed.abi7826 (2021). 10.1126/scitranslmed.abi7826 PMC9924070 34609205

[25] Güttler T (2021). Neutralization of SARS-CoV-2 by highly potent, hyperthermostable, and mutation-tolerant nanobodies. The EMBO Journal.

[26] Huo J (2021). A potent SARS-CoV-2 neutralising nanobody shows therapeutic efficacy in the Syrian golden hamster model of COVID-19. Nat. Commun..

[27] Haga K (2021). Nasal delivery of single-domain antibody improves symptoms of SARS-CoV-2 infection in an animal model. PLoS Pathog..

[28] Wrapp D (2020). Structural basis for potent neutralization of betacoronaviruses by single-domain camelid antibodies. Cell.

[29] Koenig P-A (2021). Structure-guided multivalent nanobodies block SARS-CoV-2 infection and suppress mutational escape. Science.

[30] Doremalen NV (2021). Intranasal ChAdOx1 nCoV-19/AZD1222 vaccination reduces viral shedding after SARS-CoV-2 D614G challenge in preclinical models. Sci. Transl. Med..

[31] Park J-G (2021). Immunogenicity and protective efficacy of an intranasal live-attenuated vaccine against SARS-CoV-2. iScience.

