# Supplementary material for: First-in-class trispecific VHH-Fc based antibody with potent prophylactic and therapeutic efficacy against SARS-CoV-2 and variants
Source: Sci Rep. 2022 Mar 9;12:4163. doi: 10.1038/s41598-022-07952-4 (PMC8905027; doi:10.1038/s41598-022-07952-4)
Supplement: Supplementary file 1 — Supplementary Information. [file 41598_2022_7952_MOESM1_ESM.pdf]

First-in-class trispecific VHH-Fc based antibody with potent prophylactic and therapeutic efficacy against SARS-CoV-2 and variants

Allison Titong[1], Sachith Gallolu Kankanamalage[1], Jianbo Dong[1], Betty Huang[1], Nicholas Spadoni[1], Bo Wang[1], Meredith Wright[1], Keegan L.J. Pham[1], Anh Hai Le[1], and Yue Liu[1,2,] *

1. Ab Studio Inc. 3541 Investment Blvd., Suite 3, Hayward, CA 94545, USA
2. Ab Therapeutics Inc. 3541 Investment Blvd., Suite 3, Hayward, CA 94545, USA

* Corresponding author. All inquiries should be directed to yue.liu@antibodystudio.com

Supplementary Figure legends

Figure S1: ABS-VIR-001 is well-tolerated and prevents SARS-CoV-2-luc pseudovirus infection in huACE2 mice. Tamoxifen inducible ACE-2 mice were challenged with SARS-CoV-2-luc pseudovirus (intranasally, I.N.) and were randomly divided into 3 groups and treated as indicated in Figure 1A and observed for body weight and bioluminescence (BLI). A) Body weight was indistinguishable between the treatment Groups 2 and 3 and the untreated Group 1, indicating ABS-VIR-001 was well-tolerated. (Error bars = SD) B) Raw BLI measurement form D3, D4, and D7 are shown with a BLI reduction from D3 to D7 for Group 2 and Group 3. (Error bars = SD)**.**

Figure S2: SARS-CoV-2 infection in huACE2 mice leads to a dose-dependent pathological change on lung tissue. Human ACE-2 expressing mice were challenged with SARS-CoV-2 (intranasally, I.N.) with various PFUs to determine the appropriate dose for the study. A) SARS-CoV-2 associated pathological changes on the lung tissue on mice challenged with 10 and 100 PFU. At D4, even mouse challenged with 10 PFU showed pathological changes in the lung (mice 1-1, 1-2, and 1-3), at 100 PFU there is a greater degree of pathological changes (mice 2-1, 2-2, and 2-3).

Figure S3: ABS-VIR-001 was well-tolerated in all animals within the prophylactic and treatment groups in huACE2 mice challenged with authentic SARS-CoV-2. Human ACE-2 expressing mice were challenged with SARS-CoV-2 (intranasally, I.N.), randomly divided into 4 groups and treated as indicated in Figure 2A, followed by the observation of their mean body weight and clinical symptoms. A) The quantitation of the mouse mean bodyweight normalized to the initial weight shows that all the mice within the prophylactic and treatment groups do not lose weight over time, indicating that ABS-VIR-001 is well-tolerated in animals.

Figure S4: ABS-VIR-001 maintains high binding to SARS-CoV-2 triple mutant variant. A) Binding kinetics graph for global KD of ABS-VIR-001 to wildtype (wt) RBD. B) Binding kinetics graph for global KD of ABS-VIR-001 to triple mutant (TriMut) RBD with mutations associated with

the Alpha and Beta SARS-CoV-2 variants. The data was generated by the GatorPrime software, and graphed in Prism (GraphPad). Taken together, this data shows that the mutations to the S protein RBD only show a slight decrease in the KD value which is still within picomolar level and a high level of binding.

Figure S5: ABS-VIR-001 completely blocks SARS-CoV-2 triple mutant variant. A) ABS-VIR-001 binds wt, and variants Delta, and Lambda SARS-CoV-2 blocks wt S protein RBD and Trimut S protein RBD in a comparable manner at every concentration tested, indicating that the blocking capacity is fully maintained despite the mutation. Furthermore, the VHH-Fc cocktails of the individual components of ABS-VIR-001 show a drastic, but incomplete reduction in blocking, demonstrating the overall efficiency of the trispecific ABS-VIR-001 antibody format.

Supplementary Tables

Table S1. Changes in body weight and survival of mice infected with SARS-CoV-2.

Group	Number of Mice	Infectious dose	Group weight (g), dpi					Survival number, dpi				
			0	1	2	3	4	0	1	2	3	4
1	3	10PFU	68.4	68.0	68.4	68.8	68.4	3	3	3	3	3
2	3	100PFU	69.4	68.9	68.9	67.9	42.3	3	3	3	3[a]	2[a]
3	3	330PFU	66.1	65.6	65.9	64.0	-	3	3	3	3[a]	0
4	3	1000PFU	69.1	66.8	66.9	66.1	-	3	3	3	3[a]	0
5	3	10000PFU	68.8	68.9	68.2	40.1	-	3	3	3	2[a]	0

[a] Observed depression

Table S2. Viral load and pathological changes in lung tissue of mice on day 3 (1000 PFU) or day 4 (10 or 100 PFU) after infection SARS-CoV-2.

Group	Number of mice	Infectious dose	Mouse Number	Pulmonary virus copy number [Log_{10} (Copies/g)]
1	3	10PFU	1-1	8.01
			1-2	<2
			1-3	7.59
2	3	100PFU	2-1	<2
			2-2	<2
			2-3	6.66
4	3	1000PFU	3-1	9.01
			3-2	9.71
			3-3	8.56

Table S3. Pathological changes of lung tissue in mice on day 3 (100 PFU) or day 4 (10 PFU) after infection with authentic SARS-CoV-2.

Group	Number of mice	Infectious dose	Mouse Number	Pathological changes in the lungs
1	3	10 PFU	1-1	Degeneration of bronchiolar epithelial cells, no other obvious changes
			1-2	Degeneration of bronchiolar epithelial cells, no other obvious changes
			1-3	Mild to moderate perivascular inflammation, degeneration of bronchiolar epithelial cells, and a small amount of inflammatory cell infiltration around
2	3	100 PFU	2-1	Mild perivascular inflammation, degeneration of bronchiolar epithelial cells
			2-2	Degeneration and necrosis of bronchiolar epithelial cells (nucleus condensation), inflammatory cell infiltration around blood vessels
			2-3	Mild perivascular inflammation, small blood vessel and alveolar wall capillary congestion, bronchiolar epithelial cell degeneration, perivascular edema, inflammatory cell infiltration

Supplementary Figures

Figure S1

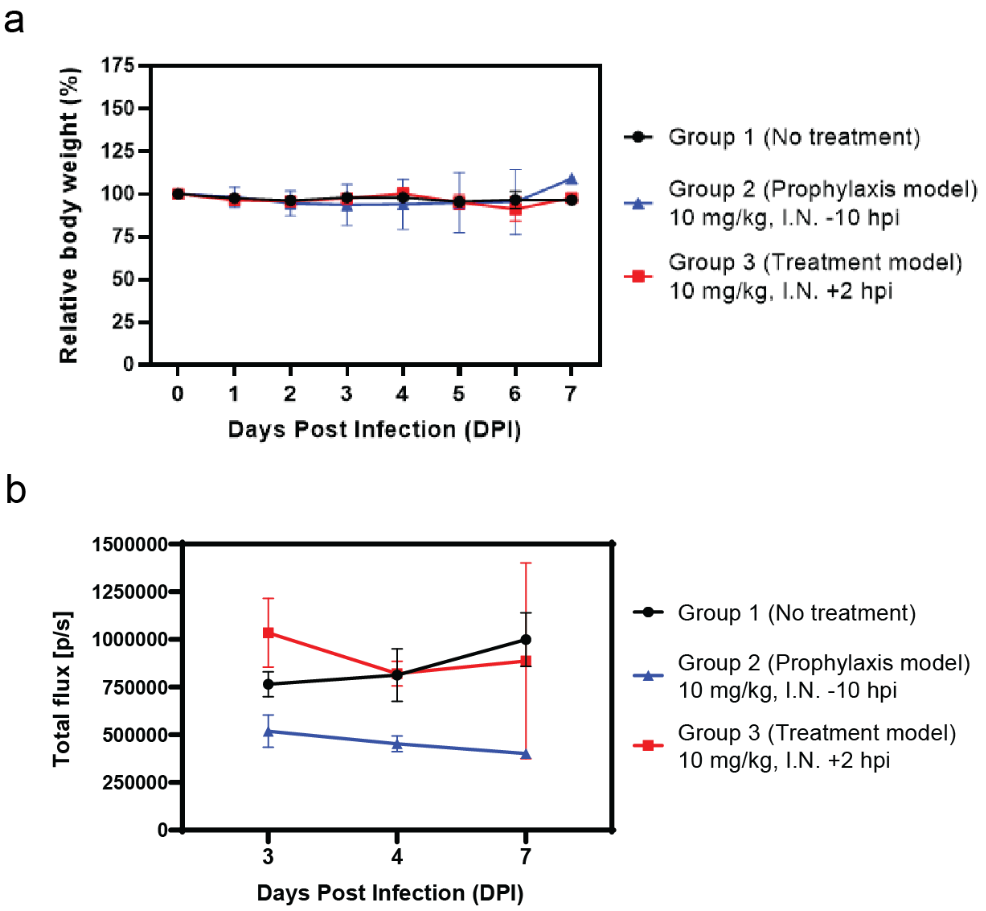

Figure S2

a

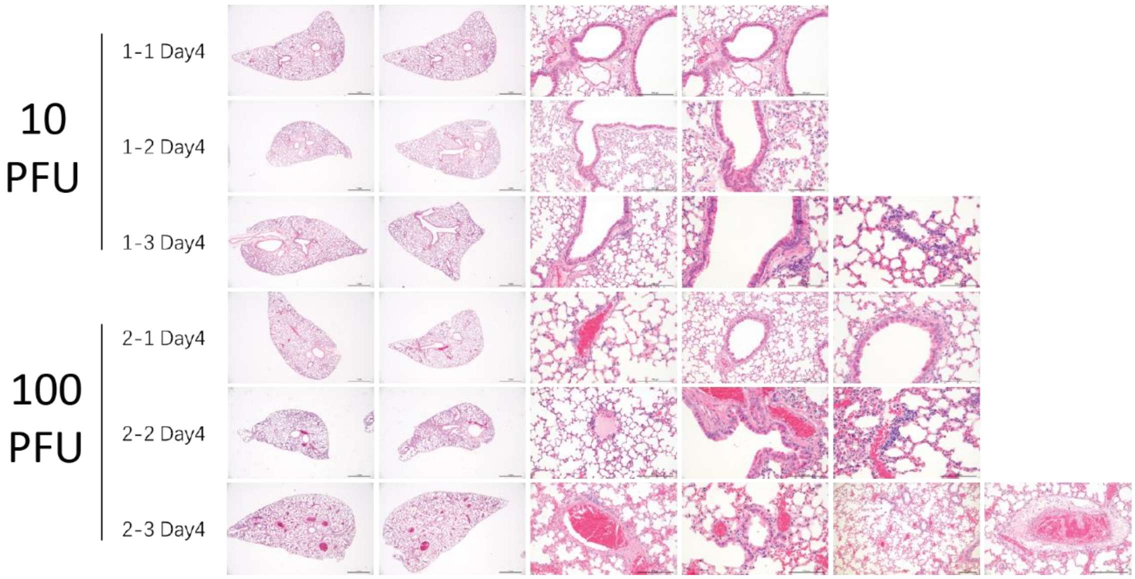

Figure S3

a

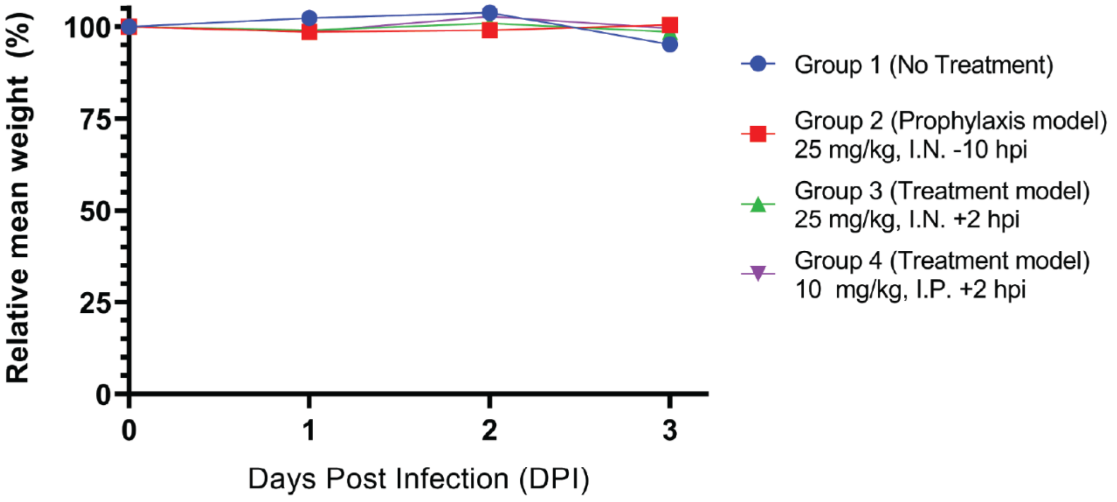

Relative mean weight (%)
100
75
50
25
0
0
1
2
3
Days Post Infection (DPI)
Group 1 (No Treatment)
Group 2 (Prophylaxis model) 25 mg/kg, I.N. -10 hpi
Group 3 (Treatment model) 25 mg/kg, I.N. +2 hpi
Group 4 (Treatment model) 10 mg/kg, I.P. +2 hpi

Figure S4

a

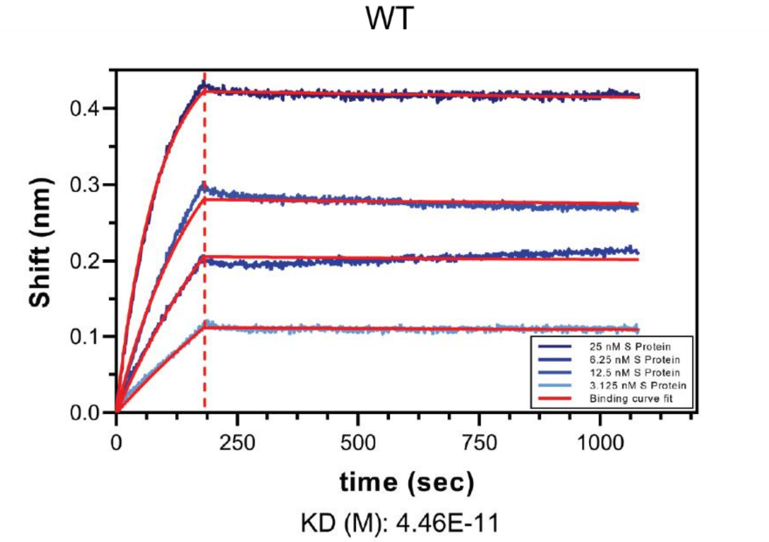
WT
Shift (nm)
0.0
0.1
0.2
0.3
0.4
0
250
500
750
1000
time (sec)
25 nM S Protein
6.25 nM S Protein
12.5 nM S Protein
3.125 nM S Protein
Binding curve fit
KD (M): 4.46E-11

b

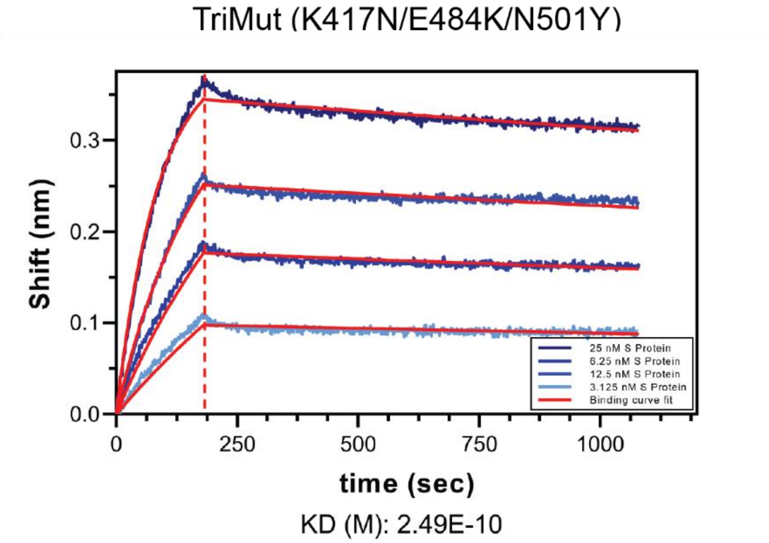
TriMut (K417N/E484K/N501Y)
Shift (nm)
0.0
0.1
0.2
0.3
0
250
500
750
1000
time (sec)
25 nM S Protein
6.25 nM S Protein
12.5 nM S Protein
3.125 nM S Protein
Binding curve fit
KD (M): 2.49E-10

170 **Figure S5**

a

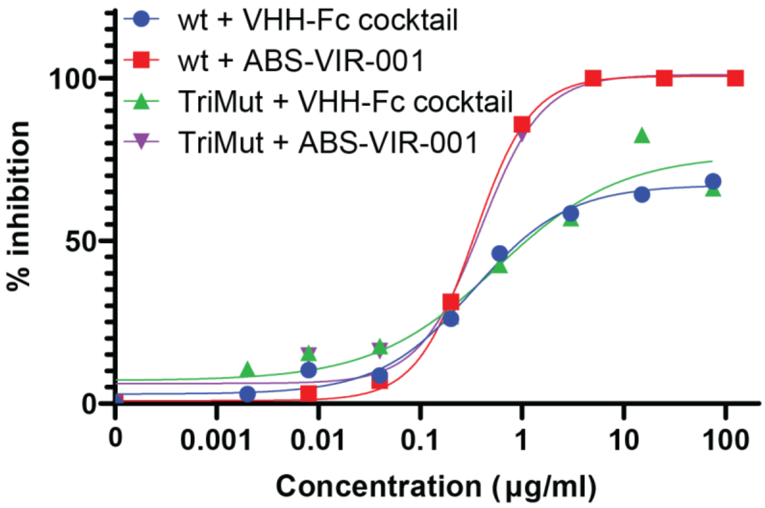

171
